# An evolutionarily conserved transcriptional response to viral infection in *Caenorhabditis* nematodes

**DOI:** 10.1186/s12864-017-3689-3

**Published:** 2017-04-17

**Authors:** Kevin Chen, Carl J. Franz, Hongbing Jiang, Yanfang Jiang, David Wang

**Affiliations:** Departments of Molecular Microbiology and Pathology and Immunology, Washington University in St. Louis, School of Medicine, 660 S. Euclid Avenue, St, Louis, MO USA

**Keywords:** Host-virus interaction, Orsay virus, Santeuil virus, *N. parisii*, *C. elegans*, *C. briggsae*, Pathogen infection, Transcriptional profile

## Abstract

**Background:**

*Caenorhabditis elegans* is a powerful model organism for probing many biological processes including host-pathogen interactions with bacteria and fungi. The recent identification of nematode viruses that naturally infect *C. elegans* and *Caenorhabditis briggsae* provides a unique opportunity to define host-virus interactions in these model hosts.

**Results:**

We analyzed the transcriptional response of pathogen infected *C. elegans* and *C. briggsae* by RNA-seq. We identified a total of 320 differentially expressed genes (DEGs) in *C. elegans* following Orsay virus infection. The DEGs of known function were enriched for ubiquitin ligase related genes; however, the majority of the genes were of unknown function. Interestingly, many DEGs that responded to Orsay virus infection were similar to those induced by *Nematocida parisii* infection, which is a natural microsporidia pathogen of *C. elegans* that like Orsay virus infects intestinal cells. Furthermore, comparison of the Orsay virus DEGs in *C. elegans* to Santeuil virus DEGs in *C. briggsae* identified 58 *C. elegans* genes whose orthologs were likewise differentially expressed in *C. briggsae*, thereby defining an evolutionarily conserved response to viral infection.

**Conclusions:**

The two different species *C. elegans* and *C. briggsae,* which diverged ~18 million years ago, share a common set of transcriptionally responsive genes to viral infection. Furthermore, a subset of these genes were also differentially expressed following infection by a eukaryotic pathogen, *N. parisii*, suggesting that these genes may constitute a broader pan-microbial response to infection.

**Electronic supplementary material:**

The online version of this article (doi:10.1186/s12864-017-3689-3) contains supplementary material, which is available to authorized users.

## Background

*Caenorhabditis elegans* is a model organism widely used to interrogate host-pathogen interactions [1, 2]. In recent years, studies in *C. elegans* have identified genes that are essential for immunity against bacterial and fungal pathogens. For instance, roles for p38 MAP kinase [3], TGF-β [4], DAF-2/DAF-16 insulin-like receptor signaling [5], and the transcription factor *zip-2* [6] have been established in protection against bacterial or fungal infections in *C. elegans*. In addition, multiple studies have dissected the *C. elegans* transcriptional response to a range of different pathogens including *Bacillus thuringiensis* [7]*, Pseudomonas aeruginosa* and *Staphylococcus aureas* [8]*, Serratia marcescens*, *Enterococcus faecalis*, *Erwinia carotovora*, and *Photorhabdus luminescens* [9], and fungal pathogens including *Drechmeria coniospora* [10], *Harposporium sp.* [11] and *Nematocida parisii* [12]. There is some overlap in the transcriptional responses to the various bacterial and fungal infections, suggesting that *C. elegans* maintains both “pan-microbial” and “microbe-specific” repertoires of pathogen response genes [13]. From the transcriptionally induced genes, some functional immune response genes have been identified and characterized.

Much less is understood about host responses in *C. elegans* to viral infection, largely due to the lack of, until recently, a natural virus capable of infecting *C. elegans*. Previous studies using artificial viral infection conditions with vaccinia virus [14], nematode cells with vesicular stomatitis virus [15, 16] or a transgenic virus replicon system (Flock house virus) [17] have demonstrated antiviral roles for the programmed cell death genes *ced-3* and *ced-4,* and RNA interference (RNAi) pathways in *C. elegans*. With the discovery of Orsay virus, the first known natural viral pathogen of *C. elegans*, RNAi and ubiquitin-mediated protection against viral infection have been described [12, 18–23].

In addition to Orsay virus, two related viruses, Santeuil and Le Blanc, were discovered in wild *Caenorhabditis briggsae* strains. Orsay virus only infects *C. elegans* while Santeuil virus and Le Blanc virus only infect *C. briggsae* [18, 24]. All three viruses have a common tissue tropism and specifically infect the intestine [25]. The identification of multiple viruses that infect two host species that diverged ~18 million years ago affords the unique opportunity to define evolutionarily conserved host responses to viral infection [26]. Furthermore, *C. elegans* can also be infected specifically in the intestine by the microsporidia *N. parisii* [27]*.* Thus, host responses to these various microbial pathogens can be compared and contrasted. In this study, to define the transcriptional response to these natural pathogens, we used high-throughput RNA sequencing (RNA-seq) to quantify the host mRNA levels following different microbial infections. Collectively, these results shed light on the host response to viral infection and provide insight into the larger context of antimicrobial defense in *C. elegans*.

## Results

### *C. elegans* transcriptional response to Orsay virus infection

To define the transcriptional changes in *C. elegans* upon Orsay virus infection, we compared RNA-seq results from infected and non-infected animals. We analyzed both the laboratory reference strain N2 as well as the *rde-1* mutant, which bears a mutation in the Argonaut protein RDE-1 that is part of the RNAi pathway. The *rde-1* mutant sustains higher levels of Orsay virus replication and accumulate ~100-fold more viral RNA compared to N2 [18] enabling us to assess the impact of more robust viral infection levels, as well as a defective RNAi pathway, on the transcriptional response. Samples were analyzed at 12 h post infection (hpi), a time by which Orsay virus protein expression is observed in most *rde-1* animals [25]. We used the edgeR package [28] to identify differentially expressed genes (DEGs) in both N2 and *rde-1* strains (n = 3 replicates for each, FDR < 0.05, Table 1). The vast majority of the DEGs were up-regulated, while a small subset of DEGs were down-regulated (Table 1, Additional file 1). Among the induced genes, up-regulation ranged between 1.8-fold to over 1000-fold compared to mock control (Additional files 1 and 2).

**Table 1:** The number of *C. elegans* differentially expressed genes (DEGs) upon different pathogen infection

	Orsay virus [N2]	Orsay virus [*rde-1*]	*N. parisii* [N2]
DEGs UP	129	277	185
DEGs DOWN	1	21	11
DEGs Shared with Orsay virus [N2]	N/A	108	108
DEGs shared with Orsay virus [*rde-1*]	108	N/A	139

Differentially expressed genes were analyzed using edgeR with 3 replicates and a FDR < 0.05 cutoff. N/A: Not applicable

**Fig. 1:**
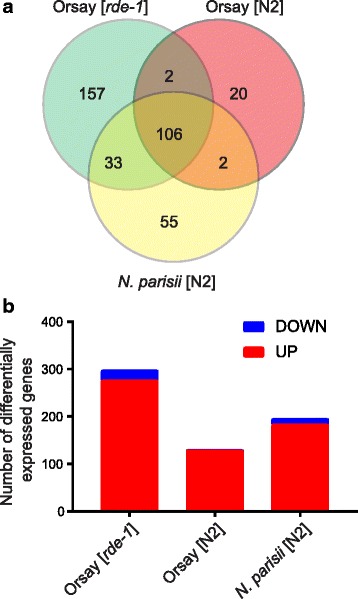
*C. elegans* differentially expressed genes upon different pathogen infections. **a** Venn diagram showed the number of genes as differentially expressed after 12 h infection for the three different conditions (Orsay virus [N2], Orsay virus [*rde-1*], and *N. parisii* [N2]) and their relationships. **b** Bar graph showed the number of genes as up-regulated or down-regulated upon different infections

Between the two different strains of *C. elegans*, there were 108 DEGs shared, while there were 22 and 190 DEGs specific to N2 and *rde-1,* respectively (Fig. 1). The majority of the DEGs were of unknown function. For the subset that had annotations, we identified several enriched gene families and functions using the software package DAVID [29, 30], (FDR < 0.05, Table 2). Both N2 and *rde-1* DEGs were enriched for several gene families including DUF38 domain genes, DUF713 domain genes, MATH (meprin-associated Traf homology) domain genes and a family of paralogs exemplified by *C17H1.3* (*C17H1 *family genes hereafter, named after the *C17H1* locus which contains the largest number of genes in this family) (Table 2). The *C17H1 *family genes have an ortholog in human, Amyotrophic Lateral Sclerosis 2 Chromosome Region Candidate 12 (ALS2CR12) which encodes a protein of unknown function. Most of the DUF38 domain containing genes also contain an F-BOX domain, which is associated with the ubiquitin ligase pathway. The DUF713 domain genes are specific to the *Caenorhabditis* genus and do not have any associated functions. Of the 22 DEGs that were specific to N2 infected with Orsay virus, no statistically enriched gene families were identified. Of the 190 *rde-1* specific DEGs, there were several additional enriched gene families including CUB-like domain genes, CUB domain genes, and zinc finger (C6HC-type) domain genes. In addition, innate immune response genes were enriched based on GO annotation (Table 2). For the DEGs that were shared between the two strains, *rde-1* DEGs in general were induced to a greater degree (Additional files 1 and 2).

**Table 2:** Gene Ontology (GO), InterPRO term enriched from *C. elegans* genes differentially expressed upon Orsay virus or *N. parisii* infection

Condition	Go term, InterPRO classification	Human ortholog	Functional annotations	Gene count	FDR
Orsay virus [N2]	**IPR026674:ALS2CR12 protein** **(** ***C17H1*** **family)**	Yes	N/A	24	6.9E-32
	**IPR002900:DUF38**	No	F-box associated	13	6.6E-09
	**IPR007883:DUF713**	No	N/A	5	1.4E-06
	**IPR002083:MATH**	Yes	MATH	8	1.3E-06
Orsay virus [*rde-1*]	**IPR026674:ALS2CR12 protein** **(** ***C17H1*** **family)**	Yes	N/A	25	2.3E-25
	GO:0045087:Innate immune response	N/A	Innate immunity	23	6.2E-13
	**IPR002083:MATH**	Yes	MATH	14	3.1E-09
	**IPR007883:DUF713**	No	N/A	7	2.9E-08
	**IPR002900:DUF38**	No	F-box associated	16	3.8E-06
	IPR003366:CUB-like domain	No	N/A	9	4.7E-06
	IPR000859:CUB domain	Yes	CUB	8	6.1E-04
	IPR002867:Zinc finger, C6HC-type	Yes	Ubiquitin related	4	2.1E-02
*N. parisii* [N2]	**IPR026674:ALS2CR12 protein** **(** ***C17H1*** **family)**	Yes	N/A	25	1.4E-29
	**IPR007883:DUF713**	No	N/A	9	1.7E-14
	**IPR002083:MATH**	Yes	MATH	11	3.0E-08
	**IPR002900:DUF38**	No	F-box associated	13	2.5E-06
	GO:0045087:Innate immune response	N/A	Innate immunity	8	5.2E-03
	IPR001841:Zinc finger, RING-type	Yes	Zinc finger	8	5.9E-03
	IPR016186:C-type lectin-like	Yes	C-type lectin-like	9	1.0E-02
	GO:0005764:lysosome	N/A	lysosome	4	2.9E-02

GO term, InterPRO classification enrichment was analyzed using online DAVID Bioinformatics Resources 6.8. **Bold** denotes conserved terms across the three infection conditions: Orsay virus [N2], Orsay virus [*rde-1*], and *N. parisii* [N2]. N/A: Not applicable

To confirm the RNA-seq results, we used quantitative real-time reverse transcription PCR (qRT-PCR) of an independent Orsay virus infection in the N2 strain to evaluate transcript levels of three highly up-regulated genes (*C17H1.3*, *C17H1.8,* and *F26F2.1*) and two genes that did not change following viral infection (*B0024.4* and *tsp-1*). All five genes yielded similar results between transcriptional profiling and qRT-PCR (Additional file 3).

### Orsay virus and *N. parisii* induced a shared transcriptional response

Because the microsporidia *N. parisii* is also an intracellular pathogen of intestinal cells in *C. elegans*, we performed a parallel transcriptional profiling of *N. parisii* infection in N2. There were 196 DEGs identified in N2 at 12 hpi of *N. parisii* (edgeR, n = 3 replicates, FDR < 0.05, Table 1); notably 108 DEGs were shared with Orsay virus infection of N2 (Fig. 1, Table 1). Thus, the majority of the Orsay virus induced DEGs in N2 were also differentially expressed following *N. parisii* infection. Another 33 DEGs were shared between *N. parisii* infection of N2 and Orsay infection of the *rde-1* strain (Fig. 1). Interestingly, only two genes were down-regulated in both *rde-1* upon Orsay virus infection and N2 upon *N. parisii* infection. The two genes, *pud-1.2,* and *pud-4*, are paralogs known to be regulated by DAF-2, an insulin-like receptor [31, 32]. Of the 55 DEGs specific to N2 infected with *N. parisii*, the enriched gene families included zinc finger (RING-type) domain genes and C-type lectin-like genes (Table 2). We compared our results with a recently published expression profile of *N. parisii* infection, which was performed in a different genetic background [12], and found the majority of the up-regulated genes that we identified were also up-regulated at 8 h post infection in the previous publication (Additional files 4 and 5).

There were four gene families enriched across all infection conditions in *C. elegans*: Orsay virus [N2], Orsay virus [*rde-1*], and *N. parisii* [N2] (Table 2). F-box domain genes (DUF38) and MATH domain genes are adapter proteins, which encode a Cullin-binding domain and a substrate-binding domain that target proteins for E3 ubiquitin-ligase mediated proteolysis [33]. There were a total of 35 unique ubiquitin ligase adaptor genes that were highly up-regulated (between 4-fold to 1000-fold), 8 of which were induced in all three conditions. Specifically, F-box proteins act in concert with other proteins that are members of the Skp/Cullin/F-box (SCF) complex to facilitate ubiquitin-ligase mediated proteolysis. Interestingly, *skr-4*, a SCF complex gene was up-regulated in all three infection conditions. Furthermore, in the *rde-1* Orsay virus infected condition, two additional SCF complex genes *skr-5* and *cul-6* were up-regulated.

**Fig. 2:**
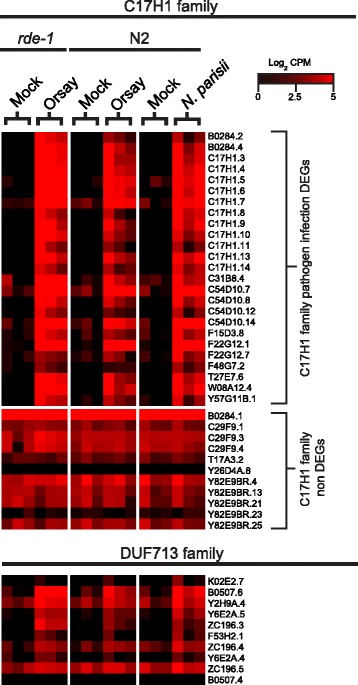
Heatmap of transcription profiles of selected gene families with multiple induced family members. The heatmap showed the log_2_ counts per million (CPM) for each gene in either *C17H1* gene family or DUF713 gene family. Each experimental condition had three replicates and each replicate was represented in a column

The *C17H1* family genes and DUF713 domain genes have no known functions. For both the *C17H1 *family genes and DUF713 domain genes, more than 50% of the family members were differentially expressed. The *C17H1 *gene family has a total of 36 members in *C. elegans,* and of those the same 25 members (except for *F22G12.7* in Orsay virus [N2] condition that was not statistically significant) were up-regulated following both Orsay virus and *N. parisii* infection (Fig. 2, Table 2). The DUF713 domain genes have a total of 10 members in *C. elegans* and have from 5–9 members of the gene family up-regulated following pathogen infection (Fig. 2, Table 2). The *C17H1* family had the most DEGs represented in the Orsay virus and *N. parisii* infections (Table 2), and some of the genes were among the highest induced with close to 1000-fold increase compared to mock infection. Given the highly distinct nature of Orsay virus from the eukaryotic microsporidium *N. parisii*, this shared transcriptional response may represent a cellular stress pathway in *C. elegans* triggered by intracellular perturbation.

### Evolutionarily conserved response to viral infection in *C. elegans* and *C. briggsae*

We next defined the DEGs in *C. briggsae* following Santeuil virus infection. Because the *C. briggsae* laboratory reference strain AF16 does not support Santeuil virus replication in our hands, we used the wild *C. briggsae* isolate JU1264 which we had previously demonstrated to be susceptible to Santeuil virus infection [18]. In *C. briggsae*, there were 258 DEGs following infection by Santeuil virus (edgeR, n = 3, FDR < 0.05, Additional file 2). Of the Santeuil virus DEGs, 37 were down-regulated and 221 were up-regulated. On a technical note, JU1264 sequence reads were mapped to the closely related AF16 reference transcriptome; strain specific sequence differences may lead to incomplete mapping to some genes and thus a potential underestimate of the DEGs.

To confirm the RNA-seq results, we used qRT-PCR of an independent Santeuil virus infection of JU1264 to evaluate transcript levels of two up-regulated genes: *CBG03198*, a gene with *C. elegans* orthologs that were also up-regulated, and *CBG06596*, an ortholog of the *C17H1 *family in *C. briggsae*. The two genes yielded similar results between transcriptional profiling and qRT-PCR (Additional file 6).

**Fig. 3:**
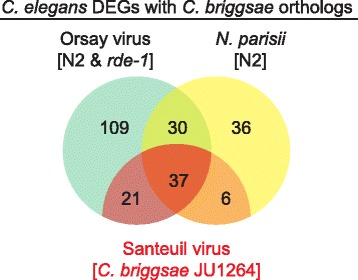
Evolutionarily conserved pan-microbial responsive *C. elegans* genes. *C. elegans* genes differentially expressed following Orsay and *N. parisii* infection that have orthologs in *C. briggsae* were differentially expressed. Red shading indicates *C. elegans* genes whose orthologs in *C. briggsae* have conserved response to Santeuil infection of *C. briggsae*

Approximately 60% of all genes in *C. briggsae* have well-defined orthologs in *C. elegans* [34]. We further compared the DEGs in *C. elegans* to their orthologous genes in *C. briggsae*. Of the 320 genes identified as differentially expressed in either N2 or *rde-1* or both (union of N2 and *rde-1* viral infection induced DEGs), 197 have orthologs in *C. briggsae*. 59 of these had *C. briggsae* orthologs that were also differentially expressed following Santeuil infection (Fig. 3, Additional file 7). The majority (57 of 59) of the DEGs were up-regulated in both species. One gene, *hmit-1.1*, was repressed in both the Orsay virus [*rde-1*] and the Santeuil virus [JU1264] conditions while *clec-7* was repressed in Orsay virus [*rde-1*] and induced in Santeuil virus [JU1264]. In total, there were 58 DEGs in the conserved response to viral infection. 29 *C. elegans* genes were induced in both N2 and *rde-1* and had corresponding *C. briggsae* orthologs induced following Santeuil infection. These, included 14 *C17H1* family genes, four DUF713 domain genes, a gene in the RNAi pathway: *C04F12.1,* and a gene downstream of *daf-16*: *dod-23*. The remaining 9 genes have no known functions. There were two *C. elegans* DEGs in N2 (but not in *rde-1*) whose orthologs in *C. briggsae* were also DEGs. These were *F14F9.3*, a zinc finger (C6HC-type) domain gene and *ZK177.8*, the human ortholog of which is SAMHD1, an antiviral gene against human immunodeficiency virus 1 [35]. Finally, there were 27 *C. elegans* DEGs in *rde-1* (but not N2) with corresponding *C. briggsae* DEGs. Six were immune related genes, four were zinc finger (C6HC-type) domain genes, three were transcription factors (*zip-1*, *zip-5* and *zip-10*), one was an RNAi related gene (*sid-*5) and the remainder had varying annotations (Additional file 7).

Interestingly, the orthologs in *C. briggsae* of the DUF38 genes and MATH domain genes that responded to both Orsay virus and *N. parisii* infection were not differentially expressed following Santeuil virus infection. This suggests that the induction of F-box and MATH genes may be a *C. elegans* specific transcriptional response. (Additional file 7).

### Evolutionarily conserved pan-microbial response

In total, 37 of the 58 conserved viral DEGs were also differentially regulated following *N. parisii* infection (Fig. 3). Strikingly, all of the 29 DEGs that were conserved between the three viral infection conditions, Orsay virus [N2], Orsay virus [*rde-1*], and Santeuil virus [JU1264], were also *N. parisii* induced DEGs. Specifically, members of the *C17H1* and DUF713 gene families appeared to be pan-microbial response genes while the zinc finger (C6HC-type) gene responses were specific to viral infection (Additional file 7). To further assess the potential roles of the conserved virus induced DEGs in the context of different pathogens, we compared our current results with previous published studies of pathogen induced host response. A prior microarray study of Orsay virus infection of *C. elegans* identified multiple DEGs [19], of which 24 DEGs were shared between the two studies in N2 background and 40 DEGs were shared in the *rde-1* mutant (Additional files 4 and 5). In addition, we identified additional statistically significant viral DEGs, possibly due to the use of synchronized animals. We examined a panel of representative bacterial pathogens including *Pseudomonas aeruginosa, Enterococcus faecalis, Staphylococcus aureus, Bacillus thuringiensis, Serratia marcescens, and Photorhabdus luminescens* [7, 8, 11, 36] and fungal pathogens including *Drechmeria coniospora* and *Harposporium sp.* [11]*.* All up-regulated and down-regulated DEGs identified from previous studies were compared to the up-regulated or down-regulated Orsay virus DEGs. *P. aeruginosa, P. luminescens* and *D. coniospora* infection each shared a significant number of DEGs with Orsay virus infection (Fisher exact test *p* < 0.001). Notably, *dod-22* was differentially expressed in all four instances. By contrast, there was an inverse association of *E. faecalis* DEGs with Orsay virus DEGs (Fisher exact test *p* < 0.001), with fewer shared DEGs than expected by chance. Finally, the other pathogens did not have a significant relationship with the Orsay virus DEGs (Table 3).

**Table 3:** Conserved responses between bacterial or fungal pathogen to Orsay virus DEGs

	Total DEGs	Bacterial/fungal DEGs shared with Orsay DEGs	Overlap significance	Number of DEGs from *C17H1* family	Number of *C17H1* DEGs shared with Orsay virus infection
*B. thuringiensis*	246	5	4.4E-01	0	0
*D. coniospora*	3787	84	6.1E-04	15	11
*E. faecalis*	3819	40	**3.7E-03**	2	2
*Harposporium sp.*	3695	60	7.1E-01	6	4
*P. aeruginosa*	146	10	1.0E-04	0	0
*P. luminescens*	3797	111	6.4E-12	12	11
*S. aureus*	386	9	2.1E-01	1	1
*S. marcescens*	3384	56	6.5E-01	11	7

Pathogen infection expression profile analysis data were obtained from [7, 8, 11, 36]. The DEGs from each pathogen were compared to Orsay virus DEGs. Association significance is calculated using Fisher exact test. **Bold** indicates a significant negative correlation

We also specifically investigated the transcriptional response of the *C17H1* gene family members following infection by other pathogens. In prior published studies, *P. luminescens* and *D. coniospora* each induced multiple of the virally up-regulated *C17H1* gene family members (Table 3). Interestingly, some of the *C17H1* family genes that were not up-regulated following viral infection were differentially expressed following infection by *D. coniospora*, *Harposporium*, *P. luminescens*, and *S. marcescens* (Table 3). Thus, there may be pan-microbial responsive C17H1 family members as well as those that respond to specific pathogens.

## Discussion

We defined the host transcriptional response to viral infection in *C. elegans* and *C. briggsae*. From our statistical analysis of Orsay virus infections in N2 and *rde-1* mutant strains, we identified a total of 320 DEGs in *C. elegans,* of which 108 DEGs were shared. In the *rde-1* Orsay virus infection, there were more DEGs compared to infection of N2. In addition, the magnitude of the transcriptional changes in *rde-1* was generally greater. One possible explanation for this observation is that the higher levels of viral infection in *rde-1* may have created a more significant perturbation from the basal state, leading to a more robust transcriptional response. Alternatively, the lack of competent RNAi in *rde-1* may have resulted in induction of a distinct, compensatory host response. One potential limitation of these studies is that Orsay, Santeuil, and *N. parisii* infection is thought to be limited to at most the 20 intestinal cells present in *Caenorhabditis* nematodes. Because our transcriptional profiling used RNA extracted from populations of entire animals (each *C. elegans* has 959 somatic cells), some transcriptional responses may have been masked by the basal level of transcription in the uninfected cells, and thus our results are likely an underestimate of the transcriptional changes occurring in the intestinal cells.

Strikingly, 108 of the N2 DEGs were also differentially expressed following infection with the microsporidium, *N. parisii* (Fig. 1). Orsay virus is a small single stranded RNA virus with a bipartite genome of 3.6 Kb and 2.6 Kb that is only known to encode three proteins [18]. By contrast, *N. parisii* has a 4.1 Mb genome and encodes more than 2000 genes [27, 37]. Despite the lack of obvious similarity between these two microbes, the fact that a significant fraction of the transcriptional response to these two pathogens overlapped suggests that *C. elegans* may have some form of a universal “stress response”. One clear commonality between the two is that they are both intracellular intestinal pathogens of *C. elegans*; in fact they are the only intracellular pathogens of *C. elegans* described to date. Thus, the conserved transcriptional response may reflect recognition of some shared intracellular perturbation. Interestingly, although some of these shared response genes are potentially involved in the ubiquitin ligase pathway, the majority of the shared response genes are largely unannotated genes of unknown function. These genes could play important roles in immunity against pathogen infection. Alternatively, it is also possible that these genes are important for pathogen infection, and that the pathogen alters the transcriptional response to facilitate infection and replication.

Many of the characterized genes induced by Orsay virus or *N. parisii* infection in *C. elegans* were genes in the ubiquitin ligase pathway. When challenged with either Orsay virus or *N. parisii*, there were 35 unique F-box related or MATH domain genes up-regulated. In addition, SCF complex genes, such as *skr-4*, were up-regulated in all *C. elegans* infections while *skr-5* and *cul-6* were up-regulated in the *rde-1* mutant infected with Orsay virus. Most of the F-box and MATH family members have sites in their substrate binding domains that are under strong positive selection and are greatly expanded in *C. elegans* in comparison to humans [33]. This suggests a possible role of ubiquitin ligase as part of the *C. elegans* host-pathogen interaction to restrict pathogen proliferation. Indeed, SCF ubiquitin ligases are demonstrated as a line of defense against infection by Orsay virus and *N. parisii* in *C. elegans* [12]. Intriguingly, none of the DEGs in *C. briggsae* were known F-box or MATH genes, suggesting that these ubiquitin ligase pathways may be a specific *C. elegans* response.

There are varying degrees of conservation between Orsay virus response genes to other pathogens of *C. elegans*. We analyzed previously published transcriptional profiling studies of infection by 8 bacterial and fungal pathogens and identified three that have a significant fraction of DEGs shared with Orsay virus infection. The three pathogens, *P. aeruginosa, P. luminescens* and *D. coniospora,* all can affect the intestine of the worm, but each does so in unique fashion. *P. aeruginosa* PA14 primarily kills by excreted toxins*, P. luminescens* colonizes the intestinal lumen, which is characterized by the appearance of cytosolic crystalline structures of unknown origin [38], and *D. coniospora* produces threadlike hyphae that penetrate and eventually kill the infected animal [39]. Other pathogens that also target the intestine such as *E. faecalis* and *S. aureus* did not have significant DEGs in common with Orsay virus infection, demonstrating a specificity of the host response. The different responses of *C. elegans* to various pathogens suggest the existence of distinct sensing and regulatory mechanisms. One potential regulatory element in response to virus infection is *drh-1*, a RIG-I like protein in *C. elegans*. Previous studies have determined that *drh-1* both acts directly as a effector in the RNAi pathway to restrict virus replication and as a sensor of virus infection critical for downstream host responses [19, 20].

Comparative analysis of the DEGs in virus infected *C. elegans* and *C. briggsae* identified 58 *C. elegans* genes whose *C. briggsae* orthologs were also differentially expressed. Of those, 29 were shared between the three conditions: Orsay virus [N2], Orsay virus [*rde-1*], and Santeuil virus [JU1264]. Strikingly, 14 of the 29 genes were members of a single gene family, the *C17H1* family genes in *C. elegans*. Induction of members of this gene family in response to viral infection was conserved in two divergent *Caenorhabditis* nematode species despite ~18 million years of host evolution. Furthermore, analysis of other published transcriptomes identified induction of* C17H1 *family genes by bacterial and fungal pathogens. The upregulation of a subset of these genes by disparate microbes such as virus, bacteria, and fungi raises the possibility that this gene family may form the core of a pan-microbial stress response. To date, there has been no reported function associated with these family members. The large number of paralogs induced following Orsay virus or *N. parisii* infection suggests the possibility of functional redundancy, which would provide a challenge in experimental testing of the functions of these genes.

## Conclusions

Our transcriptional profiling study of both virus and microsporidium infection provides insights into the host response to pathogens. We found that distinct pathogens such as Orsay virus and *N. parisii* elicited a similar set of DEGs in *C. elegans*, suggesting that these DEGs may constitute a broad pan-microbial response to infection. Additionally, within the transcriptional profile of viral infection in the two different nematode species *C. elegans* and *C. briggsae*, we found a shared set of 58 evolutionarily conserved transcriptional responsive genes to viral infection, many of which have no known function. Given the fact that diverse hosts regulate these common genes in response to distinct viral infections suggests that they play important roles. Further studies are needed to define the impact and mechanism of action of these genes on viral infection.

## Methods

### Strains

N2 and *rde-1* (WM27) were obtained from the *Caenorhabditis* Genetics Center (CGC). Isolation of wild *C. briggsae* strains JU1264 has been described [18].

### Infectious virus filtrate preparation

Orsay virus and Santeuil virus were propagated as previously described [18]. Briefly, *C. elegans rde-1* mutants were subjected to Orsay virus infection. *C. briggsae* JU1264 were subjected to Santeuil virus infection. Infected animals were subsequently collected and homogenized. The homogenate were passed through a 0.22 μm filter to obtain filtered viruses.

### Quantification of virus titer

To measure the infectious titer of viruses, we employed a method similar to tissue culture infectious dose 50% (TCID_50_) using live *C. elegans* or *C. briggsae* in wells instead of cultured cells. We were not able to measure killing of nematodes as none of the viruses were lethal. To measure infectivity in a well, we used qRT-PCR to determine whether replication of viral RNA occurred, using a criterion of Ct value of 30 and below as positive infection. Animals were synchronized and plated on 6-well plate seeded with 20 μl OP50 food. Virus filtrates were serially diluted 10-fold to 10^-8^. 20 μl of each dilution were added to a well containing animals and combined to have four total replicates per condition. Infected animals were incubated at 20 °C for three days and collected into Trizol. RNA samples were extracted using Zymo 96-well RNA extraction kit. The stock Orsay virus filtrate had a titer of 2.3 × 10^6^ TCID_50_/ml and Santeuil virus had a titer of 8.9 × 10^6^ TCID_50_/ml.

### Pathogen inoculation

Three independent infections were conducted for each strain and pathogen. Two methods were used to infect animals. 1) Uninfected *C. elegans* (N2 and *rde-1*) and *C. briggsae* (wild isolate JU1264) were synchronized by standard bleach treatment. 2,000 embryos were seeded per well into 6-well NGM plates containing 20 μl of OP50 food and maintained at 20 °C. For each condition, 18 wells were prepared and collected. 39 h after bleaching, animals (L3 stage) were inoculated with 20 μl of Orsay virus (4.0 × 10^5^ TCID_50_ /ml) for *C. elegans* or Santeuil virus (8.9 × 10^5^ TCID_50_/ml) for *C. briggsae*, or M9 buffer as control. 12 h post infection, animals were rinsed off from the wells with 1 ml M9 buffer, supernatant were removed after centrifugation and 1 ml Trizol were added. 2) Uninfected *C. elegans* (N2 and *rde-1*) were synchronized by standard bleach treatment and 20,000 embryos were added to 10 cm NGM plates seeded with 1.5 ml of OP50. 39 h after bleaching, animals (L3 stage) were inoculated with either 200 μl Orsay virus (4.0 × 10^5^ TCID_50_ /ml), *N. parisii* microsporidia (10,000 spores/animal), or homogenates of uninfected *rde-1* passed through a 0.22 μm pore filter (mock control). *C. briggsae* (JU1264) were treated similarly and infected with 200 μl Santeuil (8.9 × 10^5^ TCID_50_/ml) virus. Animals were rinsed off from the plates and harvested in Trizol at 12 h after exposure to virus, microsporidia, or mock control.

### Preparation of RNA-seq sample

Total RNA was extracted using Trizol and mRNA was subsequently enriched using OligoTex mRNA mini (Qiagen) according to the manufacturer’s protocol. RNA concentrations were assessed by Qubit fluorimeter (Life Technologies) and 10–100 ng of each sample were sent to Genome Technology Access Center in the Department of Genetics at Washington University School of Medicine (GTAC) for RNA-Seq.

### RNA-Seq Analysis

Illumina HiSeq platform-generated single end reads of 50 bp were aligned to N2 reference strain and AF16 reference strain transcriptomes (WS250) with TopHat2 [40]. The aligned reads were counted with HT-Seq [41]. Only uniquely aligned reads were counted and used for downstream analysis. The read counts were processed with R package edgeR [28, 42]. Samples were subjected to batch effect adjustment as there were two different methods of preparation and all three replicates were independently conducted on different days. The resulting counts were subjected to standard edgeR differential expressed gene analysis with a statistical cutoff of FDR < 0.05.

### Real time quantitative two-step RT-PCR (qRT-PCR) of host genes

1 μg of total RNA from each sample was treated with DNaseI (Fermentus) according to the manufacture’s protocol, purified using an RNeasy kit (Invitrogen) and then eluted in 20 μl RNase/DNase free water. cDNA synthesis was performed by using an oligo(dT) primer with thermoscript reverse transcriptase (Thermo Scientific) at 65 °C for 45 min. The synthesized cDNA was diluted 1:10 and 5 ul of the diluted cDNA was used for real time-qPCR. Real time qPCR was performed using Taqman qPCR master mix reagents (Applied Biosystem) on a ViiA7 real time PCR system (Applied Biosystem) following the manufacturer’s suggested protocol. Each analyzed gene was normalized to an internal control *cdc-42* gene and expressed as fold change of infected samples compared to mocked infected samples.

### Association of bacterial or fungal pathogen DEGs with Orsay virus DEGs

The significance of association between bacterial or fungal DEGs from previous studies and Orsay virus DEGs was measured by a Fisher exact test. Briefly, concordant (up-regulated in both or down-regulated in both) DEGs between a selected pathogen and Orsay virus were counted as the overlapped DEGs. Fisher exact test was calculated with the total non-overlapped pathogen DEGs, the non-overlapped Orsay virus DEGs and all other remaining genes (non-differentially expressed following bacterial, fungal or viral infection). Pair-wise comparison between pathogens DEGs against Orsay RNA were done for all pathogens.

## Additional files

Additional file 1: Heatmap of differentially expressed genes upon pathogen infections. The heatmap showed the expression level for all of the differentially expressed genes in the three infection conditions (Orsay virus [N2], Orsay virus [*rde-1*], and *N. parisii* [N2]). A) Log_2_ CPM of each gene presented. B) Median normalized Log_2_ CPM of each gene. Each CPM value was normalized to the median CPM for the given gene. Each experimental condition had three replicates and each replicate was represented in a column. Samples that did not have measurable expression were grey. (PDF 1297 kb)

Additional file 2: List of differentially expressed genes upon pathogen infections identified by edgeR. (XLSX 75 kb)

Additional file 3: Confirmation of *C. elegans* RNA-seq with qRT-PCR. Expression of N2 response genes to Orsay virus infection with RNA-seq was confirmed with qRT-PCR. qRT-PCR results were normalized to *cdc-42* before calculating fold-change. (PDF 29 kb)

Additional file 4: Comparison of DEGs with previous publications. Venn diagrams showed the comparison of DEGs from our current study to previous published studies. A) Comparison of up-regulated DEGs from *N. parisii* [N2] infection. The most similar conditions from *Bakowski* et al. were used (8 h and 16 h post *N. parisii* infection). B) Comparison of up-regulated genes in N2 after Orsay virus infection between current study and *Sarkies* et al. C) Comparison between down-regulated genes in N2 after Orsay virus infection between current study and *Sarkies* et al. D) Comparison between up-regulated genes in *rde-1* after Orsay virus infection between current study and *Sarkies* et al. E) Comparison between down-regulated genes in *rde-1* after Orsay virus infection between current study and *Sarkies* et al. (PDF 462 kb)

Additional file 5: List of differentially expressed genes compared to previous publications. (XLSX 24 kb)

Additional file 6: Confirmation of *C. briggsae* RNA-seq with qRT-PCR. Expression level from RNA-seq of JU1264 response genes to Santeuil virus infection was confirmed with qRT-PCR. qRT-PCR results were normalized to *cbr-cdc-42* before calculating fold-change. (PDF 28 kb)

Additional file 7: Summary of Orsay virus DEGs in *C. elegans* that have *C. briggsae* orthologs DEGs by Santeuil virus infection. (XLSX 20 kb)

## Abbreviations

ALS2CR12: Amyotrophic Lateral Sclerosis 2 Chromosome Region Candidate 12
CPM: Counts per million
DAVID: Database for Annotation, Visualization, and Integrated Discovery
DEGs: Differentially expressed genes
MATH: Meprin-associated Traf homology
qRT-PCR: Quantitative real-time reverse transcription PCR
RNAi: RNA interference
RNA-seq: RNA sequencing
SCF complex: Skp/Cullin/F-box complex
