# Supplementary figures and images for: An evolutionarily conserved transcriptional response to viral infection in Caenorhabditis nematodes
Source: BMC Genomics. 2017 Apr 17;18:303. doi: 10.1186/s12864-017-3689-3 (PMC5392922; doi:10.1186/s12864-017-3689-3)

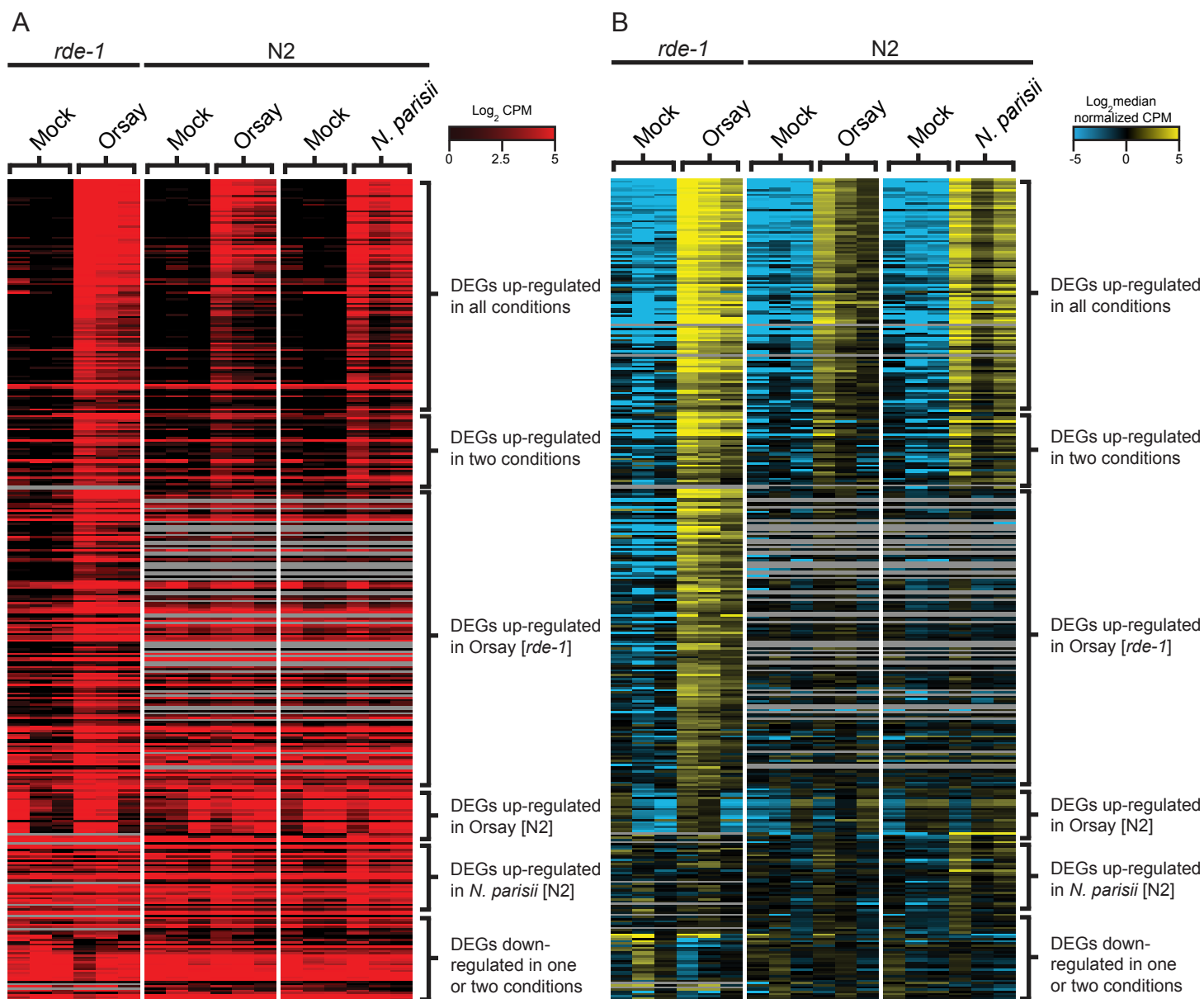

Supplement: Supplementary file 1 — Heatmap of differentially expressed genes upon pathogen infections. The heatmap showed the expression level for all of the differentially expressed genes in the three infection conditions (Orsay virus [N2], Orsay virus [rde-1], and N. parisii [N2]). A) Log2 CPM of each gene presented. B) Median normalized Log2 CPM of each gene. Each CPM value was normalized to the median CPM for the given gene. Each experimental condition had three replicates and each replicate was represented in a column. Samples that did not have measurable expression were grey. (PDF 1297 kb) [file 12864_2017_3689_MOESM1_ESM.pdf]

**Fold Change in Gene Expression  
(relative to mock infection)**

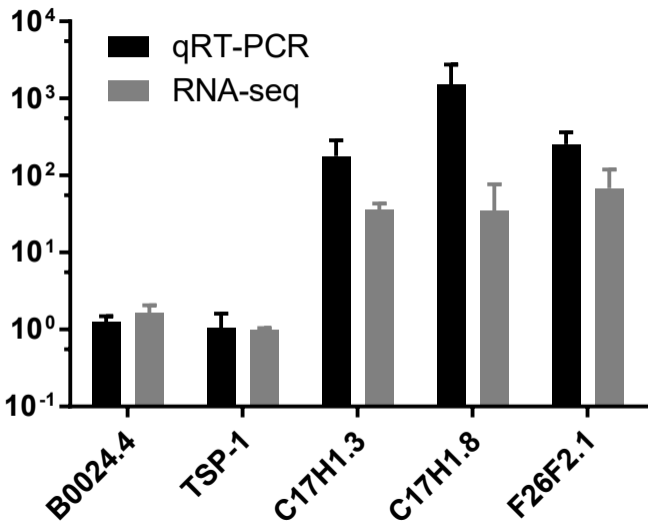

Supplement: Supplementary file 3 — Confirmation of C. elegans RNA-seq with qRT-PCR. Expression of N2 response genes to Orsay virus infection with RNA-seq was confirmed with qRT-PCR. qRT-PCR results were normalized to cdc-42 before calculating fold-change. (PDF 29 kb) [file 12864_2017_3689_MOESM3_ESM.pdf]

A

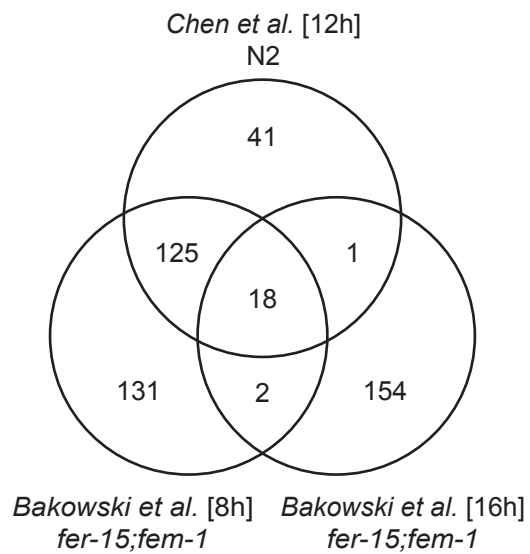

B

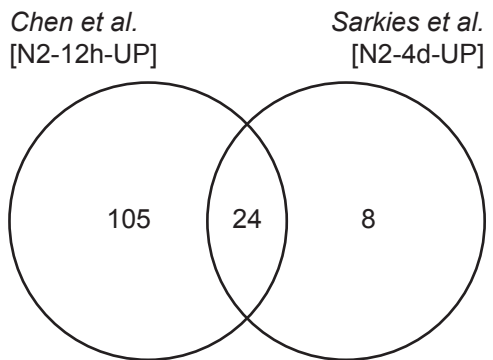

C

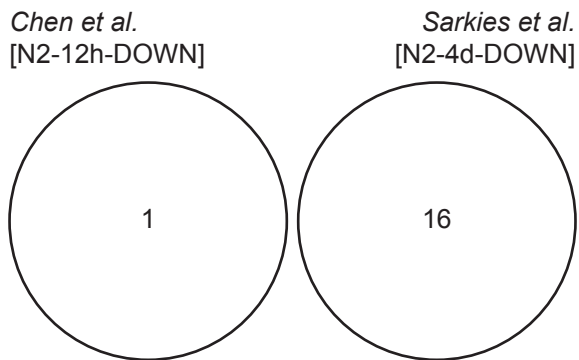

D

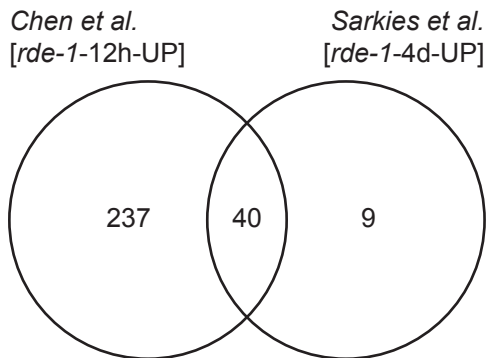

E

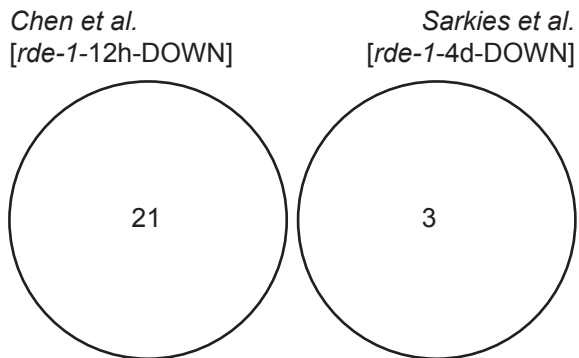

Supplement: Supplementary file 4 — Comparison of DEGs with previous publications. Venn diagrams showed the comparison of DEGs from our current study to previous published studies. A) Comparison of up-regulated DEGs from N. parisii [N2] infection. The most similar conditions from Bakowski et al. were used (8 h and 16 h post N. parisii infection). B) Comparison of up-regulated genes in N2 after Orsay virus infection between current study and Sarkies et al. C) Comparison between down-regulated genes in N2 after Orsay virus infection between current study and Sarkies et al. D) Comparison between up-regulated genes in rde-1 after Orsay virus infection between current study and Sarkies et al. E) Comparison between down-regulated genes in rde-1 after Orsay virus infection between current study and Sarkies et al. (PDF 462 kb) [file 12864_2017_3689_MOESM4_ESM.pdf]

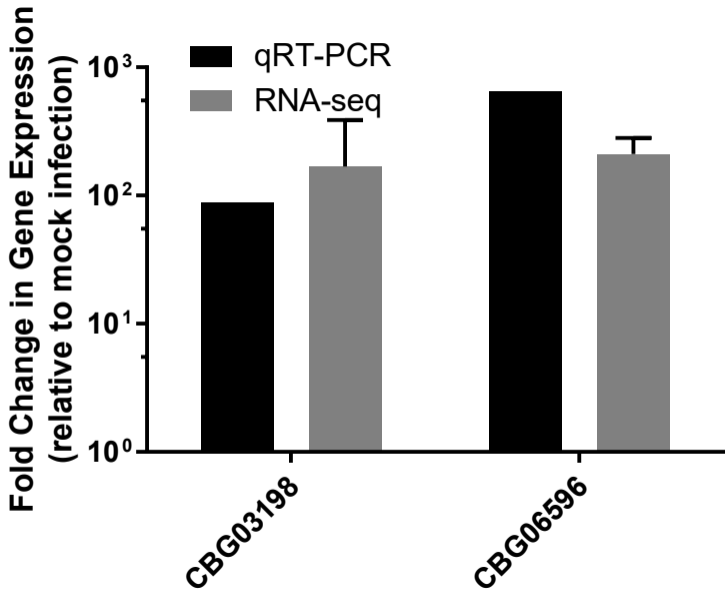

Supplement: Supplementary file 6 — Confirmation of C. briggsae RNA-seq with qRT-PCR. Expression level from RNA-seq of JU1264 response genes to Santeuil virus infection was confirmed with qRT-PCR. qRT-PCR results were normalized to cbr-cdc-42 before calculating fold-change. (PDF 28 kb) [file 12864_2017_3689_MOESM6_ESM.pdf]
